# Revisiting Thyroglobulin Measurement: Current Methods and Future Perspectives Using Dried Blood Spot Sampling for Enhanced Clinical Practice

**DOI:** 10.3390/metabo15120769

**Published:** 2025-11-27

**Authors:** Nicole Monza, Claudia Fumagalli, Lisa Pagani, Natalia Shelly Porto, Felisia Di Nicoli, Clizia Chinello, Simone Serrao, Eleonora Bossi, Marta Nobile, Fabio Pagni, Fulvio Magni, Vanna Denti

**Affiliations:** 1 Proteomics and Metabolomics Unit, Department of Medicine and Surgery, University of Milano-Bicocca, 20900 Monza, Italy; n.monza@campus.unimib.it (N.M.); claudia.fumagalli@unimib.it (C.F.); lisa.pagani@unimib.it (L.P.); n.porto@campus.unimib.it (N.S.P.); f.dinicoli@campus.unimib.it (F.D.N.); clizia.chinello@unimib.it (C.C.); simone.serrao@unimib.it (S.S.); e.bossi13@campus.unimib.it (E.B.); m.nobile13@campus.unimib.it (M.N.); fulvio.magni@unimib.it (F.M.); 2 Department of Medicine and Surgery, Pathology, University of Milano-Bicocca, Fondazione IRCCS San Gerardo dei Tintori, 20900 Monza, Italy; fabio.pagni@unimib.it

**Keywords:** thyroglobulin, liquid chromatography tandem mass spectrometry, dried blood spots

## Abstract

In contemporary medical practice, human thyroglobulin (Tg) stands out as the primary serum biomarker for detecting recurrence or persistence (presence of residual tumor) of differentiated thyroid carcinoma (DTC) in patients post-thyroidectomy. Immunoassays (IMAs) and radioimmunoassays (RIAs) have been implemented in clinical settings to gauge Tg levels. However, these methods can be unreliable because anti-thyroglobulin antibodies (Tg-Abs) and heterophile antibodies (HAs) interfere with assay binding, leading to either under- or overestimation of true Tg concentrations. Liquid chromatography tandem mass spectrometry (LC-MS/MS) has emerged as a distinctive alternative tool for Tg measurements. Despite its potential, the effectiveness of LC-MS/MS is under ongoing investigation. This review aims to provide a clear overview of existing follow-up procedures for Tg quantification and evaluate the potential of mass spectrometry (MS) in Tg analysis. The distinctive contribution of this review is the introduction of an emerging approach combining dried blood spots (DBSs) with LC-MS/MS for Tg measurement, emphasizing their translational potential for clinical follow-up of DTC patients.

## 1. Introduction

**Figure 1:**
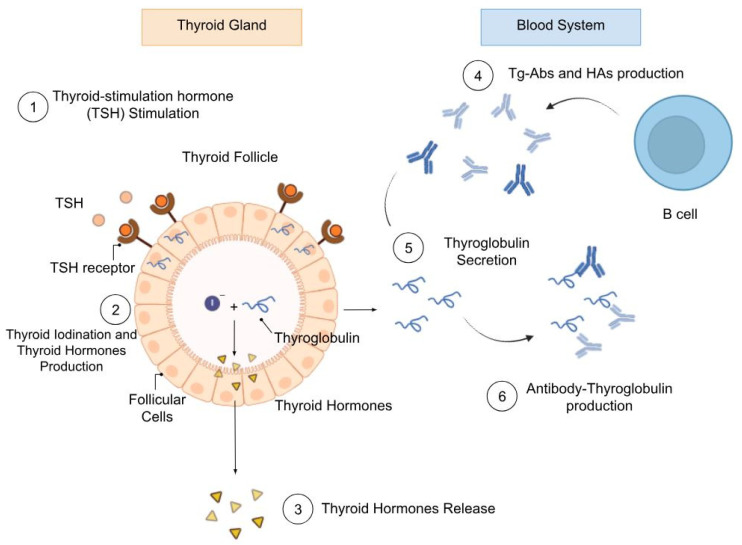
Graphical representation of the key processes involved in the synthesis, secretion, and function of Tg in the thyroid gland. Upon TSH stimulation, follicular cells are induced to produce and secrete Tg into the colloid region of the thyroid follicle (1). Within the colloid, Tg undergoes iodination, leading to the formation of thyroid hormones (2), which are subsequently released into the bloodstream (3). In addition, Tg itself can be released into circulation (5). In the blood, B cells produce Tg-Abs and HAs (4), which recognize and bind to Tg epitopes (6), potentially contributing to assay interference.

Human thyroglobulin (Tg) is a 660-kDa disulfide-linked homodimeric thyroid-specific glycoprotein essential for thyroid hormone synthesis (Figure 1) [1]. To date, according to the latest American Thyroid Association (ATA) [2] and to the European Thyroid Association (ETA) guidelines [3], Tg remains the sole blood thyroid prognostic biomarker to assess recurrence or persistence and to monitor patients with differentiated thyroid carcinoma (DTC) who underwent total thyroidectomy and ^131^I radioiodine treatment for the ablation of residual thyroid tissue [4]. While the occurrence of DTC has risen over the last ten years [5], the majority of patients, particularly those with papillary thyroid cancer (PTC), exhibit a favorable prognosis, although it can deteriorate with local invasion involving cervical lymph nodes or with distant metastases [4,5].

The basal levels of Tg in a healthy adult’s serum should range between 3–40 ng/mL [4]. Conversely, in thyroidectomized patients, who respond excellently to therapies, serum hormone therapy-Tg values should be lower than 0.2 ng/mL, or below 1 ng/mL after exogenous or endogenous thyroid-stimulating hormone (TSH) stimulation, with anti-thyroglobulin antibodies (Tg-Abs) negative in both cases. In this scenario, the risk of developing recurrences or metastatic conditions is very low, within a range of 0 to 4%. Otherwise, if therapies are not functioning adequately, the risk of recurrence increases drastically [4].

In the clinical context, venous plasma or serum Tg concentration is measured through standard immunometric assays, including radioimmunoassays (RIAs) [6] and immunoassays (IMAs) [7]. However, the presence of Tg-Abs [8] and heterophile antibodies (HAs) [9] might compromise the reliability of these methods. These antibodies bind different Tg epitopes, masking the real concentration of low-express Tg, leading to severe misdiagnoses (Figure 1). Based on the type of test, false positive or false negative results could occur. Inaccurate or delayed Tg quantification results in unnecessary treatment or in severe disease recurrence, which may worsen the patient’s condition [8,9]. Several enhancements in immunometric Tg measurement have been obtained, culminating in the development of Tg-second generation IMAs (Tg-2nd-IMAs), which have achieved significant functional sensitivity [10]. Despite these advancements, challenges related to antibody interference persist and have not been fully addressed.

In the last few years, liquid chromatography tandem mass spectrometry (LC-MS/MS) approaches have been introduced as a valid alternative in the study of Tg concentration to eliminate antibody interference issues [11,12]. Despite its potential, the effectiveness of LC-MS/MS in this context is under ongoing investigation.

Currently, the majority of clinical protein assays use liquid-form specimens (venous plasma or serum) and thus require a trained phlebotomist to collect samples. One attractive option is the use of capillary dried blood spot (DBS) specimens, which have many advantages over conventional plasma or serum sampling, including simplified sample collection procedures and increased stability that allows for more efficient shipping and storage, offering a potentially simpler, cost-effective, and minimally invasive alternative to venipuncture for Tg sampling [13]. DBS combined with MS-based tool (DBS-MS) has recently been tested in several diagnostic and clinical studies, conferring extremely promising outcomes as well as logistic and economic advantages [14,15,16,17,18].

The intended purpose of this review is to illustrate the follow-up methods used daily for Tg dosing, as well as to explore LC-MS/MS techniques coupled with enrichment protocols as a more reliable approach. In contrast to previous researches, this review presents a novel viewpoint. It outlines the primary advantages and disadvantages of serum Tg detection techniques currently used in DTC patients, and it also introduces a possible combined strategy that integrates DBS and LC-MS/MS technology. Such a strategy may improve patient follow-up, decrease analysis time and expenses, and enhance diagnostic accuracy. By bridging analytical innovation with clinical applicability, this work provides a forward-looking contribution to the field of Tg assessment.

## 2. Immunometric Assays for Thyroglobulin Measurements in Clinical Settings

In recent decades, immunometric assays have been the principal method used in clinics for the follow-up measurement of Tg levels in DTC patients post-thyroidectomy. Specifically, RIA [6], IMA [7] tests and their variants are typically employed to detect Tg on venipuncture blood samples [7,8,9]. These tests are standardized against the worldwide standard certified reference material (CRM) 457 [19,20], which consists of purified human Tg designed to be used as a primary reference material to establish calibration for immunometric tests. In the following section, the aforementioned immunometric techniques will be described. Advantages, limitations, and current issues of these techniques in clinical practice will be discussed.

### 2.1. Radioimmunoassay

**Figure 2:**
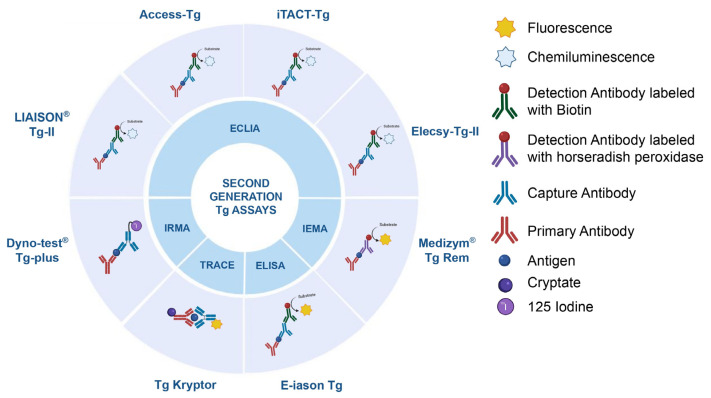
Graphical representation of the underlying mechanisms of five 2nd-Tg immunometric assays: ECLIA, IEMA, ELISA, TRACE, and IRMA. Each assay is shown in association with its corresponding commercial Tg kit: LIAISON^®^ Tg II, iTACT-Tg, Access-Tg, Elecsys^®^ Tg II (ECLIA), E-IASON Tg (ELISA), Medizym^®^ Tg Rem (IEMA), Tg Kryptor (TRACE), and Dynotest^®^ Tg-plus (IRMA). The core detection principles, capture, and signal generation strategies used in each method are highlighted. ECLIA: chemiluminescent enzyme immunoassay, ELISA: enzyme-linked immunosorbent assay, IRMA: immunoradiometric assay, TRACE: time-resolved amplified cryptate emission and EIMA: immunoenzymometric assay.

RIA was one of the earliest applicable routine methods in the diagnostic field. A clinically useful competitive Tg-RIA was first developed by A. J. Van Herle et al. in 1973 by estimating Tg concentration based on the competition between serum Tg and a Tg labeled with ^125^I, used as a reagent, for a limited amount of rabbit polyclonal Tg antibody [6]. RIA reagents contain radioisotope molecules to track antigen-antibody interaction using specific polyclonal antibodies. Among the traditional RIAs, the immunoradiometric assays (IRMAs) are applied in clinical settings [21]. Unlike RIAs, IRMAs do not rely on competitive binding, making them faster and often more sensitive. However, they require the use of two antibodies: one labeled with a radioactive isotope and one unlabeled. The radiation of the resulting complex is directly proportional to the analyte concentration (Figure 2). Moreover, with the advent of the second-generation RIAs (2nd-RIAs), the functional sensitivity of these tests has further increased. A well-established second-generation IRMA used in the research field is the Dyno-test ^®^ Tg-plus, which reaches a functional sensitivity of approximately 0.1 ng/mL and greater specificity than first-generation technologies (Table 1) [22]. Despite their high accuracy, both RIAs and IRMAs require strict handling protocols due to the use of radioactive materials, and they have largely been replaced by non-radioactive methods like IMAs in many labs [7,23].

### 2.2. Immunoassay

IMAs represent a cornerstone of modern diagnostic and research laboratories due to their remarkable specificity, sensitivity, and versatility. In this scenario, competitive RIAs have been completely replaced in clinics due to the higher sensitivity of IMAs—ten times higher compared to the RIA ones—and quicker turnaround times [4,8,38]. In the context of Tg monitoring, first-generation IMAs are completely substituted with the implementation of new Tg-2nd-IMAs [8,38]. The most common methods used include enzyme-linked immunosorbent assay (ELISA) [34,39], immunoenzymometric assay (IEMA) [40], time-resolved amplified cryptate emission (TRACE) assay [27,29,30,32], and chemiluminescent enzyme immunoassay (ECLIA) [24,25,26,28,33,34,35,36,37], all of which achieve functional sensitivity of 0.10 ng/mL or higher. The four mentioned Tg-2nd-IMAs rely on the specific Tg-antibody binding but differ in how they generate and detect the signal. ECLIA (LIAISON^®^ Tg II, iTACT-Tg, Access-Tg, Elecsy-Tg-II) and ELISA (E-iason-Tg) are highly sensitive IMAs commonly used in the diagnostic field. They are streptavidin-biotin-based double-antibody sandwich assays that use both a capture and a detection antibody to bind Tg, followed by signal generation through electrochemiluminescence (ECLIA) or enzymatic color development (ELISA), ensuring high specificity and quantitative accuracy [24,25,26,28,33,35,36,37,40]. Similarly to ELISA, IEMA (Medizym^®^ Tg Rem) uses a traditional sandwich approach, with two antibodies binding the target analyte—one immobilized, the other enzyme-linked—producing a color signal proportional to the analyte concentration after substrate addition [40]. Unlike the aforementioned techniques, TRACE (Tg Kryptor) is a homogeneous method that uses energy transfer between fluorescent labels that emit light only when brought into close proximity by antigen binding, with time-resolved detection minimizing background and enhancing sensitivity [24,27,29,30,32].

Table 1 presents seven well-established Tg-2nd-IMA kits, widely used in research and designed to produce accurate and reliable data for clinical applications.

Although both RIAs and IMAs reach a substantial functional sensitivity, several technical and biological limitations are still present, including their susceptibility to the interference effects of different types of circulating antibodies, affecting the reliability of the outcomes [8,9,10].

### 2.3. Antibody Interference

The clinical utility of Tg-IMAs monitoring for tumor persistence or recurrence in DTC patients is severely compromised by the interference of Tg-Abs and HAs, resulting in either an over- or underestimation of serum Tg concentration. Misdiagnoses due to these interferences may lead to needless therapies or severe outcomes in non-treated patients [41,42,43,44,45].

Tg-Abs occur in 15% of healthy individuals and in 20–30% of patients with DTC, targeting specific epitopes on Tg. According to the last guidelines, the longitudinal measurement of Tg and Tg-Abs blood level is recommended with the same intralaboratory IMAs [2,3]. As a result, the measured Tg-Abs concentration can serve as a stand-in tumor marker for Tg. Serum Tg-Abs measurement tests are standardized based on the international reference preparation MRC 65/93 [46]. Despite the standardization, wide variability in the outcomes and in the analytical sensitivity of these tests still remains unsolved [4]. RIAs generally demonstrate superior tolerance to such interference compared to IMAs [23], thereby facilitating more precise determination of Tg levels in DTC post-operative patients [47,48]. IMAs’ methods are notably more susceptible to antibody interference, which may result in erroneous diagnoses and less reliable Tg measurements [47,48].

Nonetheless, a novel Tg-IMA based on a fully automated ECLIA, which employs an effective sample pretreatment technique (iTACT) capable of inactivating Tg antibodies and dissociating the Tg-TgAbs complexes in samples, has been recently developed [36,37]. This method provides affordable and sensitive Tg measurement and recovery in Tg-Abs-positive samples, and it has been proved to resist Tg-Abs interference. HAs interference was not mentioned in the available studies [36,37]. Indeed, HAs non-competitive bind animal antigens or other antibodies used in IMAs [9,49,50], potentially acting as a bridge between the capture and detection antibody. Therefore, false positive results or artificially higher outcomes are detected in the absence or presence of analytes, respectively [9,48,49,50]. Human anti-mouse antibodies (HAMAs) represent the most common type of HAs produced by the human immune system in response to exposure to mouse-derived proteins or monoclonal antibodies used in therapies [51,52]. Thus, they might interfere with immunodiagnostic tests that use mouse antibodies, affecting the accuracy of the analysis [9,53,54]. In this context, strategies to minimize interference from HAMAs may include using alternative assays, blocking agents, or carefully selecting antibodies with reduced immunogenicity [52]. HAs interference affects not only the detection of serum Tg but also Tg-Abs measurement [49,50,51] and the assessment of most of the proteins and biomarkers detected through IMAs [53,54]. A comprehensive understanding of the intricate mechanisms underlying these interferences is crucial in the development of analytical methods that ensure precision and reliability in outcomes [9,50,55,56]. New alternative approaches are emerging to bypass this interference or provide complementary markers, including Tg mRNA measurements, liquid biopsies, and exosomal Tg analysis. Tg mRNA quantification in blood is Tg-Abs independent but suffers from pre-analytical variability and low reproducibility. Liquid biopsies, including circulating tumor cells, cell-free DNA, and miRNAs, offer noninvasive detection of residual or recurrent disease, even in DTC. Measuring the exosomal Tg is a particularly promising strategy since the Tg contained within exosomes is protected from antibody interference [57]. Although their proved efficiency in bypassing Tg-Abs interference, these approaches are still experimental, lacking in standardization, and requiring complex workflows.

Unfortunately, despite advancements in the implementation of new IMAs, serum Tg measurement remains susceptible to antibody interference, and nowadays this represents the most serious issue regarding the follow-up of DTC in post-operative patients [56].

## 3. State of the Art of Mass Spectrometry as a Tool for Thyroglobulin Measurements

Protein estimation across various biological samples needs to be reproducible in biomarker research. According to this, quantitative analysis of proteins as biological markers with mass spectrometry (MS) has become increasingly promising and used to the point that it is now established in the context of early disease diagnosis and follow-up in clinical research [58,59,60].

MS measures the mass-to-charge (*m*/*z*) ratio of charged particles, enabling quantification and identification of molecules in a biological sample. For this reason, MS techniques are widely applied in many scientific fields, including physics, biochemistry, chemistry, food & nutrition, and diagnostics [59,60,61]. Among other analytical techniques, MS is renowned for its high sensitivity, specificity, and capacity to provide structural details about molecules when employed in drug development, environmental research, clinical diagnostics, metabolomics, and proteomics fields [62,63]. However, even if largely used for newborn screening and for metabolite detection, its use in clinical monitoring of proteins remains underexplored [64]. Despite its better specificity compared to ordinary IMAs and its ability to overcome the antibody interference issue, MS has not fully replaced them.

The delay is primarily due to the cost of the instrument and its maintenance, which are not accessible to many research centers and hospitals. Furthermore, the availability of highly skilled professionals to conduct the analysis is essential, both for routine analyses and for addressing any instrumental issues that may arise during the studies. Additionally, while MS provides rapid results for target protein analysis, the sample preparation step is more time-consuming compared to IMAs [64]. Lastly, the limited availability of CRM for calibrating these instruments may influence the accuracy and reliability of the results [65,66,67,68,69,70].

In the context of Tg measurement, LC-MS/MS tools had confirmed certain advantages over traditional immunometric assays, yielding a more accurate value for Tg [65,66,67,68,69,70]. During the sample preparation, the tryptic digestion step allows for the cleavage of all the proteins in the biological fluid, including Tg-Abs, HAs, and their binding with Tg. Consequently, this process is expected to eliminate the antibody interference, allowing for the detection of target ionized peptides among the biological sample [65,66,67,68,69,70]. Furthermore, Tg is known to be a heavily glycosylated protein, with at least 16 N-linked glycosylation sites, which potentially mask peptide epitopes in IMAs [71,72]. LC-MS/MS analysis benefits from enzymatic deglycosylation during sample preparation via PNGase F, which removes N-linked glycans and facilitates more consistent and accurate peptide detection [72]. This additional step, combined with tryptic digestion, potentially improves analytical reliability and enhances the ionization efficiency of target peptides.

The Tg peptides with FASTA sequences VIFDANAPVAVR (VIF) and FSPDDSAGASALLR (FSP) were found to be the most abundantly ionized Tg peptides produced after trypsin proteolytic cleavage, and they were subsequently reported to be reliable target and standard peptides for Tg quantification analysis with LC-MS/MS [11,12]. According to this, N-glycosylated-Tg should not influence the targeted LC-MS/MS analysis because Tg’s signature peptides (VIF and FSP) are non-glycosylated [72].

Andrew N. Hoofnagle et al. initially introduced an analytical protocol for assessing serum Tg by combining the immunoaffinity peptide enrichment with LC-MS/MS tool [12]. Subsequent to their work, other laboratories initiated these investigations and adopted novel LC-MS/MS methods for Tg quantification, which all included the addition of an enrichment step [66,67,69,70]. In 2012 Nigel J. Clarke et al. compared an LC-MS/MS assay for Tg quantification to two established Tg-2nd-IMAs tests, highlighting the greater capability of the MS-based assay in Tg detection, particularly in Tg-Abs positive samples, where Tg-2nd-IMAs yield inaccurate results due to interferences [66]. A first implementation of sample preparation was proposed by Mark M. Kushnir et al. using a Tg enrichment process with rabbit polyclonal anti-Tg antibody and protein precipitation followed by an MS-based test for Tg monitoring, reaching a functional sensitivity of 0.5 ng/mL [67]. Although the assay generated interesting results, showing better accuracy in Tg-Abs positive serum and plasma samples, the functional sensitivity found in the present method was lower than that of Tg-2nd-IMA diagnostic tests [67]. While LC-MS/MS Tg quantification was not comparable in terms of functional sensitivity to Tg-2nd-IMAs [66,67,68], advancement in sample preparation overcame this limit. Enhanced functional sensitivity (0.02 ng/mL) was obtained from Christopher M. Shuford et al. using the LC-MS/MS system operating at microliter/minute flow rates (µLC–MS/MS) [69]. To date, this approach clearly shows the highest lower limit of quantification (LLOQ) obtained from a Tg-LC-MS/MS analysis as well as from Tg-2nd-IMAs. In terms of workflow, LC-MS/MS procedures are often more complicated and time-consuming, necessitating specialized workers and meticulous sample preparation. However, the recent development of automated sample handling and high-throughput technologies, such as the one that integrates SISCAPA^®^ (Stable Isotope Standards and Capture by Anti-Peptide Antibodies) based peptide enrichment [70,73] with robotic liquid handlers, is gradually decreasing the gap [74].

As a result, the combination of enrichment steps and the analytical sensitivity of LC-MS/MS approaches set the basis to improve Tg detection in patients’ samples compared to MS alone.

The principal features of peptide-enrichment (including the type of antibody peptide, the internal standard (IS), and the biological sample used), parameters (such as the volume of sample used, and the analytical and functional sensitivity), and disparities of the major LC-MS/MS approaches for Tg developed over the past 17 years are depicted in Table 2.

### Targeted Proteomics with Selective Peptide Enrichment

Liquid chromatography (LC) coupled to a triple quadrupole (LC-QqQ) MS working in multiple reaction monitoring (MRM) mode is the principal LC-MS/MS tool employed for targeted quantitative analysis of trace compounds in complex matrices. The QqQ analyzer consists of three quadrupoles arranged in series: the first (Q1) selects ions with a specific *m/z*, excluding all others; the second (Q2), known as the collision cell, induces fragmentation of the selected ions through a process called collision-induced dissociation (CID); and the third (Q3) acts as another mass filter, detecting only the fragments of interest. This approach provides high sensitivity and exceptional specificity, as the QqQ-MS produces a signal only when the precursor ion, selected in Q1, generates fragment ions in Q2 that are filtered through Q3 before detection [75]. For these reasons, LC-QqQ-MS is the gold standard for measuring, e.g., sex hormones, replacing traditional IMAs [76,77].

Beyond hormone quantification, this technology also finds crucial applications in targeted proteomics. In fact, the use of QqQ-MS in MRM mode enables the selective monitoring of proteotypic peptides, reaching high specificity for peptide analysis, even when the precursor protein is present at low concentrations. In this context, the limit of detection (LOD) for the representative peptide can reach the nanomolar range [78]. Nevertheless, these come with drawbacks such as longer analysis times, lower reproducibility, and limited throughput.

Moreover, the complexity of the biological matrix under investigation, whether plasma or serum, along with the type of anticoagulant employed, presents significant challenges in protein quantification via MRM [79,80]. These pre-analytical factors can significantly influence the protein composition of the sample and the efficiency of ionization. Such effects may lead to variations in the abundances of targeted proteins and compromise the comparability of results [81].

Highly abundant proteins, such as albumin and immunoglobulins, compose over 90% of the total plasma content and tend to mask low-concentration proteins, including Tg (3–40 ng/mL), which has an even lower concentration in the follow-up of DTC patients. This phenomenon is also prominent in serum, although it has a less complex protein profile compared to plasma. This difference in protein abundance can lead to matrix effects and ion suppression during MRM-based LC-MS/MS analysis, reducing sensitivity and accuracy [81,82,83]. To minimize these effects, selective protein depletion, fractionation, or enrichment strategies are frequently employed, as well as the use of isotopic standards to compensate for variations in instrumental response and improve method robustness.

To overcome these limitations, selective enrichment strategies can be employed. One effective approach is SISCAPA^®^ Technology (https://siscapa.com/company/ accessed on 26 November 2025), which allows for the specific immunoaffinity enrichment of the proteotypic peptide. SISCAPA^®^ Tg-immunoaffinity enrichment assay offers numerous advantages in conjunction with the MS-based tools compared to earlier methods, particularly regarding sensitivity, specificity, and reproducibility [70,73]. This is achieved through the use of a monoclonal antibody against the specific high-abundant Tg-peptide FSP [70,73] instead of a previously employed polyclonal antibody [12,66,67]. Moreover, it eliminates the need for μLC-MS/MS and reduces the turnaround time of the sample preparation step (~4–6 h) and the runtime of the LC-MS/MS analysis (~6–7 min) compared to no-enrichment approaches (~6–10 h and ~30–60 min per batch, respectively), improving both reproducibility and analytical throughput [71,73]. With SISCAPA^®^, a functional sensitivity of 0.15 ng/mL [70] and 0.1 ng/mL [73] was achieved, comparable to that of the Tg-2nd-IMAs, demonstrating robustness in the presence of Tg-Abs and HAs.

Several validated protocols that combine SISCAPA^®^ technology with the LC-QqQ from different vendors are available [78,84,85,86] (see Table 3). This combined approach has been established as one of the best Tg-identification methods in terms of functional sensitivity in the follow-up field [70,73].

## 4. Dried Blood Spot: A Microsampling Technique for Remote Sample Collection

Blood samples are traditionally collected through venipuncture [4]. This procedure, however, might be inconvenient for patients who require frequent monitoring. In recent years, blood microsampling (≤150 μL volumes) has emerged as an alternative to traditional venous blood sampling (exceeding 1 mL). In our latest review, the main types of microsampling devices have been summarized, focusing on their respective advantages and pitfalls [87]. This technique offers several advantages: it is minimally invasive, eco-friendly, and cost-effective and does not require healthcare personnel, enabling patients to personally collect samples at home—a significant benefit for those in remote or underserved areas with limited access to healthcare facilities—and reducing analysis costs [88]. Commercially, different types of microsampling devices for DBS, dried plasma and dried serum can be found.

Whatman^®^ 903 DBS (Cytiva, Global, Little Chalfont, UK) device was the first to come, and it is still the most widely used microsampling method. It consists of a paper-based filter card and was developed by Robert Guthrie to measure phenylalanine to diagnose phenylketonuria in newborns [89] and subsequently used in clinics for the detection of other pathologies [90,91,92,93,94]. Capillary blood is collected with a simple heel or finger prick and deposited onto the device, dried, and stored in a humidity-free environment until analysis [88].

Despite its potential, this microsampling technology shows several limitations. One of the major challenges is the impact of hematocrit (Hct), which influences blood viscosity and affects spot size and uniformity on the card. Hence, the amount of blood in the same punch of DBS is not homogeneous, influencing assays’ robustness and reproducibility as well as analyte recovery [88,95]. Moreover, following standardized guidelines for self-sampling, storage and transport of DBS to the hospital would limit pre-analytical variability, ensuring optimal quality [88,95].

To overcome the Hct bias inherent to DBS and its original workflow—where extraction is performed from a fixed-diameter subsample punched from the filter paper—the first strategy employed was to extract the entire blood-spotted area on the card. However, this approach requires spotting a fixed volume of blood, which cannot be reliably achieved without trained personnel supervising the sampling process [96].

To improve accuracy and reproducibility, volumetric adsorptive microsampling (VAMS, e.g., Mitra^®^ devices, Trajan Scientific and Medical, Melbourne, VIC, Australia), HemaPEN and other volumetric devices were developed, allowing for controlled sampling volumes independently from Htc [97]. The study of Velghe and Stove with Capitainer-B^®^ (Capitainer AB, Stockholm, Sweden) device demonstrates that this device is not affected by Htc variations, and there was no impact on the measured concentration of two blood metabolites, caffeine and paraxanthine, across 133 samples, covering a wide Hct range (18.8–55.0%) [97].

Another critical bias concerns analyte recovery, which has been addressed through the usage of an IS. The IS can be applied in different ways, with varying degrees of efficiency. A first method involves adding the IS before extraction via the extraction solvent; however, in this case, the IS is not integrated into the matrix and therefore does not correct for recovery bias. A second approach consists of co-spiking the IS directly with the blood before spotting, but this is not a practical solution. A third strategy involves pre-spotting the IS onto the filter paper before sample collection. This method has been shown to effectively correct the recovery bias [98]. Applying the IS after blood deposition but before extraction has also proven effective, although only when the IS is sprayed onto the paper [96].

Recently, Li et al. developed a novel approach called internal quantitative DBS (iqDBS), which consists of spotting a precise volume of blood onto a paper disc pre-impregnated with the IS [99]. This method demonstrated improved sensitivity and accuracy for the quantification of phenylalanine and tyrosine. Moreover, it more effectively compensates for potential degradation of the analyte, as any degradation affecting the target compound will also impact the IS. In addition, the iqDBS approach helps minimize inter-laboratory variability, thereby enhancing reproducibility and reliability of results, particularly in large-scale population screening [99].

Overall, the use of DBS offers clear economic advantages, as sample collection requires minimal equipment and training, shipping costs are lower due to classification as exempt biological specimens, and long-term storage (−80 °C) is significantly more cost-effective than plasma, owing to the minimal space required [100]. The work by Martial et al. [101] analyzes the cost-effectiveness potential of DBS, focusing on therapeutic drug monitoring (TDM) in two pediatric populations. Their findings clearly show that DBS are less expensive than traditional blood collection, both from a healthcare and a societal perspective, and highlight how the magnitude of the savings depends on the patient population (e.g., the number of hospital visits required for blood collection). For instance, in pediatric renal transplant patients, total cost reductions reached 61%.

In light of these considerations, the following sections will explore the application of DBS technology coupled with LC-MS/MS and in Tg detection, highlighting recent advancements aimed at overcoming current limitations and improving its clinical utility in patient monitoring.

### 4.1. Dried Blood Spot Microsampling and LC-MS/MS for Routine Molecular Testing

The use of DBS devices in combination with MS has become a new integrative approach to analyze and explore several molecules in the clinic, combining the previously discussed advantages of microsampling with the powerful and high-throughput MS-based tools [100]. The DBS-MS approach was first performed in 1996 by Sosnoff CS et al. to confirm immunoassay benzoylecgonine identification in blood samples from a large-scale epidemiological study of the use of cocaine during pregnancy [102,103]. Since then, DBS-MS applications have expanded significantly, initially for the investigation of newborn screening [104,105] and in the investigation of other pathologies [106,107,108,109,110,111,112]. DBS-MS is now routinely used for the detection of small molecules and metabolites [113,114,115,116,117,118,119,120], xenobiotics—for TDM [113,114,115,116,117,118,119,120]—big endogenous or exogenous peptides, as well as proteins [106,107,108,109,110,111,112,114,115,116,117,118,119,120,121,122,123,124] served as biomarkers. This evolving technology enhances both the sensitivity and specificity of analyte detection, enabling more accurate quantification across a range of biological targets [106,107,108,109,110,111,112,114,115,116,117,118,119,120,121,122,123,124]. However, despite the growing interest in DBS-MS for clinical diagnostics and therapeutic monitoring, several significant barriers still hinder its large-scale implementation in some routine healthcare settings. A major limitation concerns the need for regulatory validation across laboratories [125]. Since most DBS-MS assays are laboratory-developed tests, they must comply with rigorous regulatory frameworks, including CLIA (Clinical Laboratory Improvement Amendments) in the United States [126] and the CE-IVDR (In Vitro Diagnostic Regulation) in Europe [127]. These frameworks require extensive analytical validation, including assessments of accuracy, precision, linearity, carryover, matrix effects (for blood samples particularly), and limits of detection and quantification, as well as sample stability over time both for DBS and LC-MS/MS approaches [125]. However, the absence of standardized proficiency testing programs and external quality assessment schemes for DBS-MS methods makes it difficult to demonstrate consistent clinical performance and comparability across different laboratories [125]. The issues related to LC-MS/MS and DBS, as previously discussed in chapters 3 and 4, highlight the challenges that limit the application of this technology in the clinical field. Particularly, spot-to-spot variability due to Hct differences and volume variation of the blood upon contact with the DBS filter paper remains an important issue that could influence MS analysis and that must be addressed. Overcoming these barriers necessitates coordinated efforts among clinical laboratories, regulatory bodies, and industry partners to establish robust calibration materials, standardized operating procedures, and dedicated training programs. Such initiatives are crucial for enabling the reliable, cost-effective, and widespread clinical translation of DBS-MS technologies.

### 4.2. Dried Blood Spot Microsampling for Thyroglobulin Assays

In the context of microsampling approaches, multiple Tg analyses on DBS (Tg-DBS) have been conducted to assess thyroid function, micronutrient insufficiency, and iodine status in children and pregnant women, resulting in outstanding clinical outcomes [128,129,130,131,132,133,134,135,136]. DBS cards collect drops of blood, followed by an extraction process that varies between the laboratories, requests, and type of analysis, and finally, the sample is processed with immunological tests that use antibodies for analyte detection [131,132,133,134,135,136]. In this context, Zimmermann, M. B. et al. introduced and developed a dried whole-blood spot Tg assay adapted for the Whatman^®^ 903 device, using a two-site dissociation-enhanced lanthanide fluorescent immunoassay (DELFIA) test (PerkinElmer Life Sciences, Wallac, Turku, Finland) [128]. This assay assesses Tg as an indicator of thyroid status in goitrous children receiving iodized salt treatment. Later, the same assay was used to evaluate the role of Tg in children with an excess or deficiency of iodine intakes [129,130]. Subsequently, a new low-cost Tg-DBS enzyme-linked immunosorbent assay (Tg-DBS-ELISA) was introduced, aiming to detect a wide range of Tg concentrations for the evaluation of iodine nutrient status [132]. The same procedure was applied in other studies to check deficiency nutrition and thyroid function status in children and pregnant women [133,134]. Despite the widespread use of Tg-DBS applications, no studies to date have investigated the feasibility or diagnostic performance of DBS-based assays for detecting low-level human Tg as a biomarker for DTC recurrence. Exploring this application could offer extreme advantages for long-term surveillance of DTC patients, especially in low-resource or remote settings where venipuncture is impractical.

**Figure 3:**
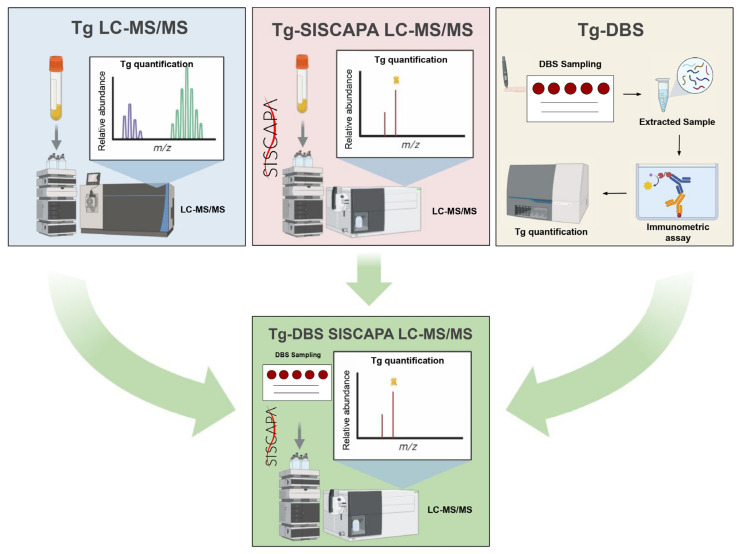
Graphical representation of the integration of DBS sampling, SISCAPA^®^ enrichment technology and LC-MS/MS tool for Tg sampling and quantification (green square). The blue square illustrates venous blood sampling followed by Tg quantification using LC-MS/MS alone. The red square shows the same approach with the addition of SISCAPA^®^ technology. The yellow square represents DBS sampling followed by Tg quantification using IMAs.

The integration of DBS with more analytically specific MS offers a novel and less invasive approach for post-treatment monitoring in DTC patients, especially considering the analytical limitations of current IMA-based methods in detecting low-level Tg. Based on these, a combined high-throughput multiplex protein assay based on the SISCAPA^®^-MS approach has been recently implemented to explore a wide range of protein biomarkers in DBS filter papers. A panel of ‘normalization proteins’ was developed and employed to improve the normalization of protein measurements in DBS in line with the volume variability and to monitor a range of clinically important biomarkers [137]. This strategy could serve as the basis for the application of high-throughput multiplex Tg-DBS SISCAPA^®^ LC-MS/MS technology (as shown in Figure 3), a state-of-the-art method that might significantly enhance analytical sensitivity through the use of magnetic beads. These antibody-linked magnetic beads are specifically aimed at target peptides through specific antibodies, providing an extremely powerful instrument for peptide detection in small sample volumes.

In line with these, a potential limitation of DBS analysis would be the substantially lower available sample volume per spot (5–10 μL plasma equivalent) compared with conventional SISCAPA^®^-MS assays using hundreds of μL of serum or plasma. This reduction may affect assay sensitivity, but the high specificity of antibody-based enrichment and recent improvements in MS detection and microflow chromatography can compensate for the smaller input. Furthermore, the ability to conduct longitudinal DBS sampling enables repeated measurements, which contributes additional robustness, even in light of the lower per-spot volumes.

Finally, the implementation of this approach (Figure 3) would combine the advantages of Tg-LC-MS/MS in eliminating Tg-Abs interference, with an effective protein enrichment protocol (SISCAPA^®^) and with the benefit of non-invasive and remote sample collection (DBS).

## 5. Conclusions and Future Directions

The evolving landscape of DTC monitoring calls for more accessible, sensitive, and patient-centered tools. Conventional serum Tg-2nd-IMAs, while clinically valuable, are limited by pre-analytical challenges, Tg-Abs and HAs interference, and the need for frequent hospital-based sampling [65,66,67,68,69,70]. MS has been introduced as a strong and accurate technology that could be employed for measuring Tg levels, providing healthcare professionals with useful information for thyroid cancer diagnosis, monitoring, and therapy [65,66,67,68,69,70]. The integration of LC-MS/MS methods with protein enrichment techniques, including SISCAPA^®^ technology (Figure 3), has further improved Tg quantification in human biological samples, enhancing assay sensitivity [70,73].

Moreover, the integration of DBS sampling with LC-MS/MS offers the possibility to decentralize Tg monitoring, allowing for remote sample collection outside of clinical settings [128,129,130,131,132,133,134,135,136]. Periodic at-home DBS sampling mailed to a central laboratory could enable continuous Tg surveillance in long-term follow-up, with minimal patient burden, facilitating rapid clinical response to recurrence while supporting individualized patient care [88]. However, the clinical translation of DBS-MS for Tg quantification will benefit from focused efforts in several areas. Future research should focus on establishing standardized protocols to ensure inter-laboratory comparability of DBS–MS results, including harmonization of calibration materials, extraction efficiencies, and analytical thresholds. Automation of DBS sample processing and data analysis could further enhance throughput and reproducibility, paving the way for routine clinical adoption. Additionally, the development of multi-analyte panels that integrate Tg with complementary biomarkers, such as Tg-Abs, Tg mRNA, or metabolomic profiles, could offer a more comprehensive molecular signature for DTC recurrence and treatment response.

In line with these observations, the possibility of remote sampling through DBS coupled with immunoenrichment-based Tg LC-MS/MS detection might open unprecedented opportunities not only for regular surveillance in thyroidectomized patients (Figure 3), but also for translational research. In addition to targeted and quantitative Tg measurements, further proteomic analyses can be conducted on the residual material from the same sample, facilitating the simultaneous examination of multiple markers. By integrating clinical diagnostics and advanced research capabilities, this approach paves the way for a new era in onco-endocrinology—one where remote, sensitive, and data-rich monitoring strategies can truly aid personalization of patient treatment. Collaborative validation studies between academic and clinical laboratories will be necessary to connect proof-of-concept with widespread use, which will ultimately improve precision monitoring in thyroid cancer care. Together, these strategies are expected to facilitate broader adoption of Tg-DBS SISCAPA^®^-LC-MS/MS in clinical practice and support its use for personalized patient management.

## Figures and Tables

**Table 1:** Principal immunometric assays available.

Year	Kit	Manufacturer	Method	CRM 457	Sample	LOQ	LOD
2007 [24]	Access Tg	Beckman Coulter, Brea, CA, USA	ECLIA	Yes	Serum	0.1 ng/mL	NA
2011 [25]							NA
2012 [26]							0.01 ng/mL
2016 [27]							NA
2018 [28]							0.05 ng/mL
2011 [25]	E-iason	Iason, Graz-Seiersberg, Austria	ELISA	Yes	Serum	0.03–0.04 ng/mL	NA
2011 [22]	Dyno-test ^®^ Tg-plus	BRAHMS GmbH, Hennigsdorf, Germany	IRMA	Yes	Serum	0.1 ng/mL	0.05 ng/mL
2016 [27]	Tg-Kryptor	BRAHMS GmbH, Hennigsdorf, Germany	TRACE	Yes	Serum	0.15 ng/mL	0.09 ng/mL
2017 [29]							NA
2019 [30]							NA
2019 [30]							NA
2021 [31]							NA
2022 [32]							NA
2021 [33]	LIAISON^®^ Tg II	DiaSorin, Saluggia, Italy	ECLIA	Yes	Serum	0.10 ng/mL	NA
2021 [34]							0.057 ng/mL
2021 [34]	Medizym^®^ Tg Rem	Medipan, Blankenfelde-Mahlow, Germany	EIMA	Yes	Serum	0.09 ng/mL	0.026 ng/mL
2021 [34]	Elecsy-Tg-II	Roche Diagnostic, Rotkreuz, Switzerland	ECLIA	Yes	Serum	0.10 ng/mL	0.04 ng/mL
2023 [35]							NA
2021 [36]	iTACT-Tg	Fujirebio Inc., Tokyo, Japan	ECLIA	Yes	Serum	0.03 ng/mL	NA
2023 [37]					Serum, Plasma		

Summary of the principal immunometric kits used in clinical applications. Manufacturers, type of analysis, functional and analytical sensitivity are reported. NA: not available, LOQ: limit of quantification, LOD: limit of detection, CRM: certified reference material 457, ECLIA: chemiluminescent enzyme immunoassay, ELISA: enzyme-linked immunosorbent assay, IRMA: immunoradiometric assay, TRACE: time-resolved amplified cryptate emission and EIMA: immunoenzymometric assay.

**Table 2:** Recent reported LC-MS/MS methods for the quantification of Tg in human serum and plasma.

Year	Antibody Peptide	IS	Sample	Volume	Reference Material	LOD	LLOQ	Additional Method Aspects
2008 [12]	Polyclonal (rabbit) anti-VIF	tSIL Peptide	Serum	100 μL	CRM 457	2.6 ng/mL	2.6 ng/mL	NA
2012 [66]	Polyclonal (chicken) anti-VIF	tSIL Peptide	Serum	100 μL	NA	0.3 ng/mL	0.4 ng/mL	NA
2013 [67]	Polyclonal (rabbit) anti-VIF	cSIL Peptide	Serum and Plasma	500 μL	NA	0.25 ng/mL	0.5 ng/mL	Protein Precipitation
2020 [69]	Monoclonal anti-FSP	cSIL Peptide	Serum	400 μL	NA	0.0057 ng/mL	0.02 ng/mL	Micro-flow chromatography
2022 [73]	Monoclonal anti-FSP	tSIL Peptide	Serum	400 μL	Husky Ref	NA	0.1 ng/mL	SISCAPA^®^ Technology
2022 [70]	Monoclonal anti-FSP	tSIL Peptide	Serum	400 μL	Husky Ref	NA	0.15 ng/mL	Mobile Phase DMSO-SISCAPA^®^ Technology

Description of the principal LC-MS/MS based methods for Tg quantification, reporting the antibody peptides, the internal standards, the type of biological sample, and the reference material used. The amount of volume, LOD, and LLOQ detected are depicted. NA: not available, IS: Internal Standard, LLOQ: lower limit of quantification, LOD: limit of detection, DMSO: dimethyl sulfoxide.

**Table 3:** Vendors reported technical notes of LC-QqQ-MS methods coupled with SISCAPA^®^ Technology for the quantification of Tg in human serum and plasma. NA = not available.

Vendors	LC System	MS System	SISCAPA^®^ Tg Peptides
Waters Corporation [78]	ACQUITY UPLC I-Class PLUS FL System	Xevo TQ Absolute Mass Spectrometer	FSP
Waters Corporation [84]	ACQUITY UPLC M-Class	Xevo TQ-S operating in MRM Mode with Unit Mass Resolution	FSP
Sciex [85]	NA	Citrine Triple Quad MS/MS System	FSP
Agilent Technologies [86]	Agilent 1290 Infinity LC	Agilent 6490 Triple Quadrupole MS with iFunnel technology	FSP and VIF

## Data Availability

No new data were created or analyzed in this study.

## Abbreviations

The following abbreviations are used in this manuscript:

ATA	American Thyroid Association
Tg-Abs	Anti-thyroglobulin antibodies
CRM	Certified reference material
ECLIA	Chemiluminescent enzyme immunoassay
CID	Collision-induced dissociation
DTC	Differentiated thyroid carcinoma
DELFIA	Dissociation-enhanced lanthanide fluorescent immunoassay
DBS	Dried blood spot
DBS-MS	Dried blood spot MS-based tool
ELISA	Enzyme-linked immunosorbent assay
ETA	European Thyroid Association
FSP	FSPDDSAGASALLR
Hct	Hematocrit
HAs	Heterophile antibodies
HAMAs	Human anti-mouse antibodies
IMAs	Immunoassays
IEMA	Immunoenzymometric assay
iqDBS	Internal quantitative DBS
IS	Internal standard
µLC–MS/MS	LC-MS/MS system operating at microliter/minute flow rates
LOD	Limit of detection
LOQ	Limit of quantification
LLOQ	Lower limit of quantification
LC-MS/MS	Liquid chromatography tandem mass spectrometry
LC-QqQ	Liquid chromatography triple quadrupole
*m/z*	Mass-to-charge ratio
MRM	Multiple reaction monitoring
PTC	Papillary thyroid cancer
RIAs	Radioimmunoassays
2nd-RIAs	Second generation RIAs
SISCAPA^®^	Stable isotope standards captured by anti-peptide antibodies
Tg-DBS	Tg analyses on DBS
Tg-DBS-ELISA	Tg-DBS enzyme-linked immunosorbent assay
Tg-2nd-IMAs	Tg-second generation IMAs
TDM	Therapeutic drug monitoring
Tg	Thyroglobulin
TSH	Thyroid-stimulating hormone
TRACE	Time-resolved amplified cryptate emission
UHPLC	Ultrahigh-performance liquid chromatography
VIF	VIFDANAPVAVR
